# A System of Medicine

**Published:** 1880-02

**Authors:** 


### BIBLIOGRAPHICAL.

A SYSTEM OF MEDICINE. Edited by J. Russell Reynolds, M.D., F.R.S., Fellow of the Royal College of Physicians of London, Fellow of the Imperial Leopold-Carolina Academy of Germany, etc., etc.; with numerous additions and illustrations by Henry Hartshorne, A.M., M.D., Fellow of the College of Physicians of Philadelphia, formerly Professor of Practice of Medicine in Medical Department of Pennsylvania College, etc., etc. In three volumes. HENRY C. LEA: Philadelphia. 1879.

The two volumes of the above work that have been placed before us by the efficient agents, Messrs. Chambers & Co., of this city, have impressed us very favorably with the value and usefulness of the publication. The more recent improvements and advances in the science and art of medicine are elaborately and scientifically presented to the reader. Special and general pathology are fully discussed, and all the advances in treatment, prophylaxis and hygiene comprehensively presented. As a text-book to the student, it is elaborate and comprehensive; as a book of ready reference to the practitioner it is invaluable, and as a work embodying the views of the highest British Medical authority, it promises to equal any publication of the kind extant. Vol. I is devoted to general diseases and diseases of the nervous system, while Vol. II. contains treatises on diseases of the respiratory and circulatory systems. We hope at some future time to give a more extended notice of the publication, but time and space prevent that consideration which we are satisfied the work deserves.

